# Commentary: The roles of serum vitamin D and tobacco smoke exposure in insomnia: a cross-sectional study of adults in the United States

**DOI:** 10.3389/fnut.2024.1500041

**Published:** 2024-12-04

**Authors:** Xiangming Li, Peixing Huang, Guizhong Huang

**Affiliations:** Shantou Hospital of Traditional Chinese Medicine, Guangzhou University of Traditional Chinese Medicine, Shantou, Guangdong, China

**Keywords:** NHANES, data, question, vitamin D, tobacco smoke exposure

We read with great interest the article titled “The roles of serum vitamin D and tobacco smoke exposure in insomnia: a cross-sectional study of adults in the United States” by Gao et al. (1). The authors utilized data from the NHANES database and highlighted the potential regulatory role of serum vitamin D (VD) levels in the association between tobacco smoke exposure and insomnia. Their work offers valuable insights for the early identification and prevention of insomnia in high-risk populations. We commend the authors for shedding light on this important topic, which has potential implications for health policy.

However, we would like to raise a specific concern regarding the data used in the study. The authors mentioned that they utilized the NHANES data from 2005 to 2008. Upon review, we found that the data on serum 25(OH)D levels from the 2007 to 2008 NHANES cycle are currently unavailable for download (as stated on the NHANES website: https://wwwn.cdc.gov/nchs/nhanes/continuousnhanes/default.aspx?BeginYear=2007). We kindly request clarification on how the authors were able to access these data.

In addition, while we acknowledge the authors' comprehensive approach in controlling for various covariates (e.g., smoking, depression, OSA, work status, work shifts, and VD collection season), we would like to highlight some limitations of the study. As the study was based on a cross-sectional design, it could not establish causal relationships between VD levels, cotinine, and insomnia. Additionally, it could not conclusively determine the regulatory role of VD in the association between cotinine and insomnia.

We also wish to highlight that the study's reliance on self-reported insomnia might have introduced recall bias. Although clinical guidelines suggest that insomnia is often diagnosed through patient history rather than polysomnography (PSG), self-reported data can be subject to inaccuracies. In addition, the dietary intake information collected via two 24-h recalls might have further contributed to recall bias.

Furthermore, we would like to expand on the geographical limitations of the study. The findings from a U.S.-based cohort may not be directly applicable to regions with different seasonal and geographical characteristics. For instance, in tropical areas with only two distinct seasons, factors such as sunlight exposure, which affect vitamin D synthesis, may differ significantly from those in temperate climates. We recommend that the authors discuss this limitation and caution against extrapolating these findings to populations in regions with different climates and seasonal variations.

Moreover, the U.S. population sample used in this study had specific dietary patterns that may not align with those of populations in other countries. As dietary habits can significantly influence vitamin D levels, the conclusions drawn from this study may not be generalizable to regions with different dietary patterns. We encourage the authors to address this limitation and suggest future studies exploring these relationships in populations with varying dietary intake patterns.

We emphasize the need for additional prospective studies to better elucidate the causal relationship between VD and insomnia, as well as the potential role of VD in modulating the effects of tobacco smoke exposure on sleep disturbances.

Once again, we appreciate the authors' valuable contributions and look forward to seeing further research on this topic.
